# Supplementary material for: Increasing liver stiffness is associated with higher incidence of hepatocellular carcinoma in hepatitis C infection and non-alcoholic fatty liver disease–A population-based study
Source: PLoS One. 2023 Jan 24;18(1):e0280647. doi: 10.1371/journal.pone.0280647 (PMC9873178; doi:10.1371/journal.pone.0280647)
Supplement: S1 Table — (DOCX) [file pone.0280647.s001.docx]

**S1 Table. ICD and CPT codes used for patient identification.**

CPT for TE: 91200

ICD9s for HCV: '070.41', '070.44', '070.51', '070.54', '070.70', '070.71'

ICD10s for HCV: 'B18.2', 'B19.2'

ICD9s for NAFLD: '571.8'

ICD10s for NAFLD: 'K76.0', 'K76.89', 'K75.81'

ICD9s for HepB: '070.2', '070.3'

ICD10s for HepB: 'B16' , 'B19.0', 'B19.1'
