# Supplementary material for: Increasing liver stiffness is associated with higher incidence of hepatocellular carcinoma in hepatitis C infection and non-alcoholic fatty liver disease–A population-based study
Source: PLoS One. 2023 Jan 24;18(1):e0280647. doi: 10.1371/journal.pone.0280647 (PMC9873178; doi:10.1371/journal.pone.0280647)
Supplement: S2 Table — (DOCX) [file pone.0280647.s002.docx]

**S2 Table.** **Baseline demographic data in those who did and did not develop HCC**.

|  | **Diagnosed Hepatocellular Carcinoma**  (n=538) | **No Diagnosed Hepatocellular Carcinoma** (n=39,790) |
| --- | --- | --- |
| **Age** (mean +/- standard deviation) | 64.7 +/- 10.9 | 60.6 +/- 10.9 |
| **Gender** |  |  |
| Male | 620 (99%) | 51758 (94%) |
| **Race** |  |  |
| White | 332 (53%) | 31115 (57%) |
| Black | 251 (40%) | 19369 (35%) |
| Hispanic | 50 (8%) | 3996 (7%) |
| **Risk Factors** |  |  |
| Hepatitis C Virus | 567 (90%) | 28671 (52%) |
| Hepatitis B Virus | 60 (10%) | 3674 (7%) |
| Non-Alcoholic Fatty Liver Disease | 97 (15%) | 17724 (32%) |
| Tobacco Use | 170 (27%) | 8669 (16%) |
| Alcohol Use | 171 (27%) | 10764 (20%) |
| Hypertension | 424 (68%) | 29924 (54%) |
| Dyslipidemia | 184 (29%) | 20034 (36%) |
| Diabetes Mellitus | 229 (36%) | 15011 (27%) |
