# Supplementary material for: Increasing liver stiffness is associated with higher incidence of hepatocellular carcinoma in hepatitis C infection and non-alcoholic fatty liver disease–A population-based study
Source: PLoS One. 2023 Jan 24;18(1):e0280647. doi: 10.1371/journal.pone.0280647 (PMC9873178; doi:10.1371/journal.pone.0280647)
Supplement: S3 Table — (DOCX) [file pone.0280647.s003.docx]

**S3 Table. Baseline laboratory data in those who did and did not develop HCC.**

|  | ***Diagnosed Hepatocellular Carcinoma*** *(n=538)* | ***No Diagnosed Hepatocellular Carcinoma*** *(n=39790)* |
| --- | --- | --- |
| Platelets count x10^9^ /L Median (IQR) | 156 (118.5,205) | 204 (163,250) |
| Total bilirubin (mg/dL)  Median (IQR) | 0.7 (0.5,1) | 0.6 (0.4,0.8) |
| AFP (ng/dL)  Median (IQR) | 6.6 (3.6,26.8) | 3.4 (2.3,5.2) |
| INR  Median (IQR) | 1.1 (1,1.2) | 1 (1,1.1) |
