# Supplementary material for: Increasing liver stiffness is associated with higher incidence of hepatocellular carcinoma in hepatitis C infection and non-alcoholic fatty liver disease–A population-based study
Source: PLoS One. 2023 Jan 24;18(1):e0280647. doi: 10.1371/journal.pone.0280647 (PMC9873178; doi:10.1371/journal.pone.0280647)
Supplement: Attachment — Submitted filename: Response To Reviewers.docx [file pone.0280647.s004.docx]

Journal Requirements:

When submitting your revision, we need you to address these additional requirements.

1. Please ensure that your manuscript meets PLOS ONE's style requirements, including those for file naming. The PLOS ONE style templates can be found at
   [https://journals.plos.org/plosone/s/file?id=wjVg/PLOSOne_formatting_sample_main_body.pdf](https://urldefense.com/v3/__https:/journals.plos.org/plosone/s/file?id=wjVg*PLOSOne_formatting_sample_main_body.pdf__;Lw!!IuDQRY6mOWG9IIqcpA!XthPEbZxtsmdkYQGteyD7kc-ZwQldUHfUBmD-ZwtZ0r1ps2C8RrIcMBmK_L5mQ7yMgxA-nPiO0jZ4Eip1jGYbszp5A$) and [https://journals.plos.org/plosone/s/file?id=ba62/PLOSOne_formatting_sample_title_authors_affiliations.pdf](https://urldefense.com/v3/__https:/journals.plos.org/plosone/s/file?id=ba62*PLOSOne_formatting_sample_title_authors_affiliations.pdf__;Lw!!IuDQRY6mOWG9IIqcpA!XthPEbZxtsmdkYQGteyD7kc-ZwQldUHfUBmD-ZwtZ0r1ps2C8RrIcMBmK_L5mQ7yMgxA-nPiO0jZ4Eip1jEZ_BmBxA$)

Updated style and file naming to meet PLOS one requirements.

2. Please amend your current ethics statement to include the full name of the ethics committee/institutional review board(s) that approved your specific study.

Changed within the body of the manuscript to reflect approval through the VA Northeast Ohio System Research Office and the VA Innovation and Research Review System.

3. In ethics statement in the manuscript and in the online submission form, please provide additional information about the patient records/samples used in your retrospective study. Specifically, please ensure that you have discussed whether all data/samples were fully anonymized before you accessed them and/or whether the IRB or ethics committee waived the requirement for informed consent. If patients provided informed written consent to have data/samples from their medical records used in research, please include this information.

Data was collected and stored with full Protected Health Information (PHI) on VA servers behind a VA fire wall. For data analysis, identifiers were removed and only coded data was analyzed. Given that data is de-identified and fully anonymized, the IRB committee waived the requirement for informed consent and HIPPA Authorization. The manuscript was updated to reflect this.

4. We note that the grant information you provided in the ‘Funding Information’ and ‘Financial Disclosure’ sections do not match.

When you resubmit, please ensure that you provide the correct grant numbers for the awards you received for your study in the ‘Funding Information’ section.

Grant info: UH Cleveland Medical Center, Seidman Cancer Center- small internal award for large database studies. Oracle Grant PTAEO: 17572.01.P0534.xxxxx.49275. When we resubmit, the funding info/financial disclosures will all reflect this.

5. Thank you for stating the following in your Competing Interests section:

None of the authors reports conflict of interest

Please complete your Competing Interests on the online submission form to state any Competing Interests. If you have no competing interests, please state "The authors have declared that no competing interests exist.", as detailed online in our guide for authors at [http://journals.plos.org/plosone/s/submit-now](https://urldefense.com/v3/__http:/journals.plos.org/plosone/s/submit-now__;!!IuDQRY6mOWG9IIqcpA!XthPEbZxtsmdkYQGteyD7kc-ZwQldUHfUBmD-ZwtZ0r1ps2C8RrIcMBmK_L5mQ7yMgxA-nPiO0jZ4Eip1jFF3E01Pg$) 
 This information should be included in your cover letter; we will change the online submission form on your behalf.

This was added to the cover letter in a competing interests section.

6. Please include captions for your Supporting Information files at the end of your manuscript, and update any in-text citations to match accordingly. Please see our Supporting Information guidelines for more information: [http://journals.plos.org/plosone/s/supporting-information.](https://urldefense.com/v3/__http:/journals.plos.org/plosone/s/supporting-information.__;!!IuDQRY6mOWG9IIqcpA!XthPEbZxtsmdkYQGteyD7kc-ZwQldUHfUBmD-ZwtZ0r1ps2C8RrIcMBmK_L5mQ7yMgxA-nPiO0jZ4Eip1jH7eaRVjw$) 

Added.

Reviewers' comments:

Reviewer's Responses to Questions

**Comments to the Author**

1. Is the manuscript technically sound, and do the data support the conclusions?

The manuscript must describe a technically sound piece of scientific research with data that supports the conclusions. Experiments must have been conducted rigorously, with appropriate controls, replication, and sample sizes. The conclusions must be drawn appropriately based on the data presented.

Reviewer #1: Yes

Reviewer #2: Yes

Reviewer #3: Yes

2. Has the statistical analysis been performed appropriately and rigorously?

Reviewer #1: Yes

Reviewer #2: Yes

Reviewer #3: Yes

3. Have the authors made all data underlying the findings in their manuscript fully available?

The [PLOS Data policy](https://urldefense.com/v3/__http:/www.plosone.org/static/policies.action*sharing__;Iw!!IuDQRY6mOWG9IIqcpA!XthPEbZxtsmdkYQGteyD7kc-ZwQldUHfUBmD-ZwtZ0r1ps2C8RrIcMBmK_L5mQ7yMgxA-nPiO0jZ4Eip1jFdm4jqlw$) requires authors to make all data underlying the findings described in their manuscript fully available without restriction, with rare exception (please refer to the Data Availability Statement in the manuscript PDF file). The data should be provided as part of the manuscript or its supporting information, or deposited to a public repository. For example, in addition to summary statistics, the data points behind means, medians and variance measures should be available. If there are restrictions on publicly sharing data—e.g. participant privacy or use of data from a third party—those must be specified.

Reviewer #1: Yes

Reviewer #2: Yes

Reviewer #3: Yes

4. Is the manuscript presented in an intelligible fashion and written in standard English?

PLOS ONE does not copyedit accepted manuscripts, so the language in submitted articles must be clear, correct, and unambiguous. Any typographical or grammatical errors should be corrected at revision, so please note any specific errors here.

Reviewer #1: Yes

Reviewer #2: Yes

Reviewer #3: No

Not sure of the specific concerns from reviewer #3 but any further concerns addressed below.

5. Review Comments to the Author

Please use the space provided to explain your answers to the questions above. You may also include additional comments for the author, including concerns about dual publication, research ethics, or publication ethics. (Please upload your review as an attachment if it exceeds 20,000 characters)

Reviewer #1: This is a retrospective study assessing TE stiffness relation with HCC in patients with HCV or NASH. The following suggestions should be considered:
1. In the abstract it should be stated the duration of the mean followed up for patients.

Added to abstract.

2. To include p-value for characteristics difference in table 1.

P values added to table 1.

3. To include a paragraph on the effect of HCV treatment on the population as part of the discussion.

In the third paragraph of the discussion section “HCC in the HCV subgroup”, we discuss the potential effects of HCV treatment on fibrosis. We also added a line about how treatment of HCV has been previously shown to reduce the risk of HCC and corelate with our results.

Reviewer #2: The authors analyzed the rates of HCC in both HCV and NAFLD patients. They concluded that HCC incidence increased with higher TE liver stiffness measures in both HCV and NAFLD. The experiments were conducted properly. The conclusions was solidly based on the data analyzed.

Reviewer #3: In this manuscript, Davitkov et al. present the findings of increased incidence of hepatocellular carcinoma in non-alcoholic fatty liver disease (NAFLD) and hepatitis C virus (HCV) infected patients. Overall, the paper is well written and grammatically correct. However, the clear rationale for conducting the study and the novelty is lacking. There are concerns about the results which require addressing before the manuscript can be considered for publication.

The rationale for the study is, to date, there is not data on the risk for HCC (and thus whether surveillance is needed) in non-cirrhotic patients with advanced fibrosis. This was made more clear in the revised manuscript.

Results:
1. In Table 1, there are too many gridlines in the table. For example, you have divided the heading “Race” into 4 rows including white, black, other, and unknown. You need to hide the gridlines between each group, indent, and merge the 4 subheadings into one row. This will make the table easier for readers to read. Moreover, the abbreviations in the table should be defined in the footnotes.

Fixed gridlines to make table appear cleaner. Abbreviations now in legend.

2. If I am not mistaken, your study population is divided into 2 study groups, HCV and NAFLD. Increased transient elastography was shown, indicating an increased incidence of HCC.
Do you have data which shows that patients with both HCV and NAFLD have a higher incidence rate ratio for developing HCC?

This data was not collected – in the NAFLD population, those with HCV were excluded. Though no specific exclusion was made for those with hepatitis C who may have had concurrent NAFLD, no specific data on patients with both pathologies was collected.

3. Considering your study results, I recommend changing your research title by adding both HCV and NAFLD to your article title. This will enable readers to easily understand why liver stiffness can result in HCC.

Added.

6. PLOS authors have the option to publish the peer review history of their article ([what does this mean?](https://urldefense.com/v3/__https:/journals.plos.org/plosone/s/editorial-and-peer-review-process*loc-peer-review-history__;Iw!!IuDQRY6mOWG9IIqcpA!XthPEbZxtsmdkYQGteyD7kc-ZwQldUHfUBmD-ZwtZ0r1ps2C8RrIcMBmK_L5mQ7yMgxA-nPiO0jZ4Eip1jEgGqZF5Q$)). If published, this will include your full peer review and any attached files.


If you choose “no”, your identity will remain anonymous but your review may still be made public.


**Do you want your identity to be public for this peer review?** For information about this choice, including consent withdrawal, please see our [Privacy Policy](https://urldefense.com/v3/__https:/www.plos.org/privacy-policy__;!!IuDQRY6mOWG9IIqcpA!XthPEbZxtsmdkYQGteyD7kc-ZwQldUHfUBmD-ZwtZ0r1ps2C8RrIcMBmK_L5mQ7yMgxA-nPiO0jZ4Eip1jFGyZFS0Q$).

Reviewer #1: No

Reviewer #2: No

Reviewer #3: No

[NOTE: If reviewer comments were submitted as an attachment file, they will be attached to this email and accessible via the submission site. Please log into your account, locate the manuscript record, and check for the action link "View Attachments". If this link does not appear, there are no attachment files.]

While revising your submission, please upload your figure files to the Preflight Analysis and Conversion Engine (PACE) digital diagnostic tool, [https://pacev2.apexcovantage.com/](https://urldefense.com/v3/__https:/pacev2.apexcovantage.com/__;!!IuDQRY6mOWG9IIqcpA!XthPEbZxtsmdkYQGteyD7kc-ZwQldUHfUBmD-ZwtZ0r1ps2C8RrIcMBmK_L5mQ7yMgxA-nPiO0jZ4Eip1jFh5uPiyg$). PACE helps ensure that figures meet PLOS requirements. To use PACE, you must first register as a user. Registration is free. Then, login and navigate to the UPLOAD tab, where you will find detailed instructions on how to use the tool. If you encounter any issues or have any questions when using PACE, please email PLOS at [figures@plos.org](mailto:figures@plos.org). Please note that Supporting Information files do not need this step.
