# Supplementary material for: Increasing liver stiffness is associated with higher incidence of hepatocellular carcinoma in hepatitis C infection and non-alcoholic fatty liver disease–A population-based study
Source: PLoS One. 2023 Jan 24;18(1):e0280647. doi: 10.1371/journal.pone.0280647 (PMC9873178; doi:10.1371/journal.pone.0280647)
Supplement: Attachment — Submitted filename: Response To Reviewers (2).docx [file pone.0280647.s005.docx]

Reviewer #1: Thanks to the authors for following previous suggestions. All my previous comments have been addressed in the current draft.

Reviewer #3: Results:
1. In Table 1, you put the description of abbreviations and statistical methods in the title. Please transfer this part to the footnote following Table 1. Besides, in Table 1, you missed the units for many variables. For example, “Age at TE” should be “Age at TE (years).”

Reply: Thanks for your suggestion. We have moved the abbreviations to a footnote following table 1. In the footnote, we also added explanations of abbreviations: kPa and kg/m^2^. In addition, we added units to “Age at TE” and “BMI at TE”.

2. In Table 2, please spell out the terms that the abbreviations stand for (ETOH, BMI, DM, etc.) in the footnote.

Reply: Thanks for your suggestion. We added a footnote following table 2 with abbreviations.

3. In addition, we would appreciate your responses indicated by “Reply: Thanks for your suggestions or Thanks for ……” Otherwise, we cannot easily recognize where your point-by-point answers begin.

Reply: Thanks for this note. All replies to this revision request are formatted this way.

4. In your supplementary Table S1, what does “%” indicate in “ICD9s for HCV: '070.41%…’”? ICD9 or ICD10 codes do not use the % symbol.

Reply: Thanks for your suggestion. We have removed the percent symbols – they were likely a vestige of the way the data was extracted from the database.

5. Anyway, I hope you can improve the editing quality of the table or figure design. I suggest reviewing some previous published articles for examples

Reply: Thanks for your comment. I reviewed recent articles and changed the formatting/text of all of the tables. As far as the figures, I’m not sure what would increase the editing quality, but happy to adjust anything as needed!
